# Clinical significance of P-glycoprotein and glutathione *S*-transferase π expression in gallbladder carcinoma

**DOI:** 10.3892/etm.2014.1472

**Published:** 2014-01-02

**Authors:** QING-JIU MA, YU-CUN ZHANG, JING-SEN SHI, GUO-CAI LI

**Affiliations:** 1 General Surgery Department, GaoXin Hospital of Xi’an Jiao Tong University, Xi’an, Shaanxi 710075, P.R. China; 2 Hepatobiliary Department, First Hospital of Xi’an Jiao Tong University, Xi’an, Shaanxi 710061, P.R. China

**Keywords:** gallbladder cancer, P-glycoprotein, glutathione *S*-transferase, metastasis, prognosis

## Abstract

P-glycoprotein (P-gp) and glutathione *S*-transferase π (GST-π) are not only drug-resistance markers, but also prognostic markers of various cancers. The aim of the present study was to investigate the clinical significance of P-gp and GST-π in gallbladder carcinoma (GBC). Tissue samples from 42 patients with GBC were immunostained. Demographic, clinical and follow-up data were collected and analyzed. The positive expression rates of P-gp and GST-π in the GBC tissues were significantly higher (76.2 and 64.3%, respectively) than that of chronic cholecystitis specimens (30 and 20%, respectively) (P=0.014 and 0.035, respectively), and correlated with the Nevin stage of GBC. Multivariate analysis demonstrated that patients with positive expression of P-gp and GST-π showed a significantly lower 2-year survival rate (11.1 and 12%, respectively) compared with patients with negative expression (55.6 and 45.5%, respectively) (P=0.013 and 0.036, respectively). P-gp was also found to be an independent prognostic marker of 2-year survival rate by logistic regression analysis (B=−2.76, P=0.061). Results of this study suggest that P-gp is a prognostic marker of GBC and the detection of P-gp and GST-π may contribute to the prognosis of GBC and the application of chemotherapy as a therapeutic treatment.

## Introduction

Gallbladder cancer (GBC) is a common and lethal cancer with a poor prognosis due to its insensitivity to radioactive and chemical therapies. The 5-year survival rate of GBC is <5% (1,2). Early tumor resection is the only effective and potentially curative treatment. However, a number of patients present with GBC in the later stages when surgical intervention is no longer effective. Therefore, the identification of a biomarker with diagnostic and prognostic values is crucial for the treatment of GBC.

P-glycoprotein (P-gp) is encoded by the multidrug resistance 1 (MDR1) gene and is a membrane glycoprotein. It acts as an energy-dependent drug efflux pump and is important in pharmacokinetics. Overexpression of P-gp is a major mechanism of drug resistance (3), thus, P-gp is used as a biomarker of drug resistance during the systemic treatment of various malignancies (4). In previous years, studies have identified that the expression of P-gp correlates with tumor progression and the prognosis of breast, colon and lung cancers (5–8)

Glutathione *S*-transferase π (GST-π) is a subclass of GSTs, a polymorphic supergene family of detoxification enzymes involved in the metabolism of numerous potential carcinogens (9). GST-π is mainly distributed in the placenta, lung, kidney, liver and red blood cells at a low levels of expression (10). In addition, GST-π is the most important phase II drug-metabolizing enzyme and is involved in the metabolism and detoxification of environmental carcinogens and chemotherapeutics. Previously, it was reported that the expression levels of GST-π correlate with the prognosis of malignances, including glioblastoma and breast, prostate and colorectal cancer (9,11–13).

However, the clinical significance of P-gp and GST-π in the progression and prognosis of GBC remain unknown. Therefore, the present study aimed to investigate the expression levels and prognostic values of P-gp and GST-π in GBC.

## Materials and methods

### Samples

In total, 42 tissue samples of GBC were obtained from patients at the First Hospital of Xi’an Jiao Tong University (Xi’an, China) between January 2000 and July 2005. Following excision, samples were paraffin-embedded. The diagnosis of GBC was established by histopathological analysis and surgery was performed according to the stage defined by the Nevin classification (14). In addition, 10 tissue samples obtained from patients with chronic cholecystitis were used as the controls.

Demographic and clinical data, including age, gender, the presence of gallstones and disease history, were obtained. The patient cohort included 26 females and 16 males and the mean age at surgical resection was 60.81±1.30 years. All patients did not present with complications, such as hypertension, diabetes and chronicle hepatitis. Histological types of GBC were classified according to previous studies (15,16), which included adenocarcinoma (not otherwise specified), papillary adenocarcinoma, adenosquamous, mucinous, adenocarcinomas and undifferentiated carcinoma.

This study was approved by the Ethical Committee of Xi’an JiaoTong University. All patients received oral and written information regarding the study protocol and signed an informed consent prior to inclusion in the study.

### Immunohistochemistry

Paraffin-embedded tumor tissues were sliced into 5-μm thick sections and mounted on glass. Slides were deparaffinized and rehydrated in 10 min through a graded alcohol series to deionized water in 1% Antigen Unmasking Solution (Vector Laboratories, Burlingame, CA, USA) and microwaved to enhance antigen retrieval. Tissue samples were sequentially incubated with anti-mouse immunoglobulin coupled to horseradish peroxidase (HRP). Slides were incubated with the specific primary anti-GST-π (ab47709; Abcam, Cambridge, UK) and anti-P-gp (P7965; Sigma, St. Louis, MO, USA) monoclonal antibodies with an HRP-conjugated secondary antibody (A1293; Sigma), and then stained with 3,3-diaminobenzidine and counterstained with hematoxylin and eosin. In addition, 10 tissue samples from patients with chronic cholecystitis obtained by cholecystectomy were used as the control group. Two pathologists independently observed and interpreted the results of the immunohistochemical staining.

### Assessment of staining

Staining of P-gp and GST-π was evaluated according to the percentage of positive cells under an optical microscope (Leica Microsystems, GmbH, Wetzlar, Germany; magnification, ×20). Staining intensity was classified as the following: Negative (−), no immunopositive staining or <10% of positive cells observed; weak to moderate (+), 10–30% positive cells; and high (++), >30% positive cells.

### Patient follow-up

Patients were advised to undergo 2-year regular follow-ups following GBC diagnosis. Follow-up data from 36 patients were obtained and the remaining data were lost.

### Statistical analysis

Statistical analysis was performed using SPSS software (version 11.5; SPSS, Inc., Chicago, IL, USA). Differences of expression rate among groups were analyzed by Pearson’s χ^2^ test. The Fisher’s exact test was used to assess the differences between the positive rates when the number of total cases was <40. All statistical tests were two-sided. To elucidate the risk factors for prognosis (2-year survival rate), multivariate analysis was performed using the logistic regression model. P<0.05 was considered to indicate a statistically significant difference.

## Results

### Expression levels of P-gp and GST-π in GBC

**Figure 1:**
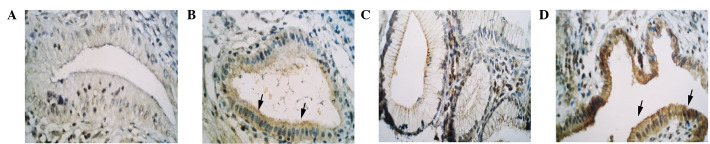
Immunohistochemical analysis of expression of P-gp and GST-π in GBC tissues. (A) Negative expression of P-gp; (B) Positive expression of P-gp was observed in gallbladder carcinoma. The majority of positive cells were located on the surface of the mucosa; C) Negative expression of GST-π in gallbladder carcinoma; (D) Positive expression of GST-π in gallbladder carcinoma (magnification, ×20). Arrows denote positive cells. P-gp, P-glycoprotein; GST-π, glutathione *S*-transferase π; GBC, gallbladder carcinoma.

**Figure 2:**
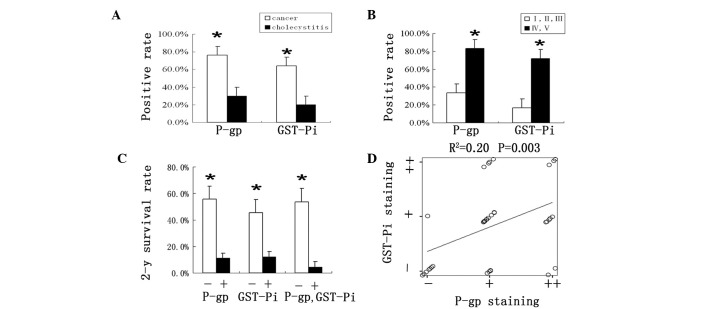
Expression levels of P-gp and GST-π correlate with metastasis and prognosis. (A) Comparison of P-gp and GST-π expression levels between GBC and cholecystitis tissues (^*^P<0.05, vs. cholecystitis). (B) Comparison of P-gp and GST-π expression levels between GBC and the early Nevin stage (I, II and III) and late Nevin stage (IV and V). *P<0.05 compared with early Nevin stage (I, II and III). (C) Differences in the 2-year survival rate between GBC tissues with a positive or negative expression of P-gp and GST-π. *P<0.05 compared with positive specimen. (D) Correlation between the expression of P-gp and GST-π. Dots denote each sample and the line represents the regression line. P-gp, P-glycoprotein; GST-π, glutathione *S*-transferase π; GBC, gallbladder carcinoma; R: correlation coefficient.

P-gp and GST-π were mainly expressed in the cytoplasm or membrane of GBC cells (Fig. 1). The positive expression rate of P-gp and GST-π in the GBC tissues were 76.2 and 64.3%, respectively, which was significantly higher than that in the chronic cholecystitis tissues (30 and 20%, respectively) (P=0.014 and P=0.035, respectively) (Fig. 2A). The expression levels were not correlated with gender, age, pathology, presence of gallstones and histological grading (Table I).

### Expression levels of P-gp and GST-π correlate with the Nevin stage

The expression levels of P-gp and GST-π in the early Nevin stages of GBC (I, II and III) were lower (33.3 and 16.7%, respectively) than that in the later stages (IV and V) (83.3 and 72%, respectively) (P=0.021 and 0.016, respectively) (Fig. 2B). As Nevin staging is classified by tumor metastasis, multivariate analysis was performed using the logistic regression model and found that P-gp staining is an independent risk factor for metastasis of GBC (R^2^=3.09; P=0.044) (Table II).

### P-gp and GST-π expression positively correlates with the prognosis of GBC

According to follow-up data of 36 cases, the expression levels of P-gp and GST-π significantly correlated with the 2-year survival rate. The 2-year survival rate in P-gp-negative patients (55.6%) was higher than that in the P-gp-positive patients (11.1%) (P=0.013). Similarly, 2-year survival rate in the GST-π-negative patients (45.5%) was also higher than that in the GST-π-positive patients (12.0%) (P=0.036). In addition, coexpression of P-gp and GST-π demonstrated the lowest 2-year survival rate (4.3%) (P=0.001) (Fig. 2C). P-gp was also found to be an independent prognostic marker of the 2-year survival rate by logistic regression analysis (R^2^=−2.76, P=0.061) (Table III).

### Correlation between P-gp and GST-π

A significant positive correlation was also found between the expression levels of P-gp and GST-π in tumor tissues (R^2^=0.20; P=0.003) and the coexpression rate was 59.5% (Fig. 2D).

## Discussion

In the present study, expression levels of P-gp and GST-π in malignant lesions were found to be higher than that of benign lesions, indicating multiple drug resistance of GBC. In addition, majority of positive cells were located on the mucosal surface of the gallbladder, this phenomena was consistent with the role of P-gp as a multidrug transporter and support the mechanism of GBC. Similar results have also previously been identified suggesting that P-gp is substantially expressed on the biliary surface of hepatocytes and small biliary ductules (17).

Although P-gp and GST-π correlate with drug resistance, their mechanisms and drug-resistant spectrum are different. GST-π is regulated *in vivo* by reactive oxygen species and its induction represents part of an adaptive response mechanism to chemical stress caused by electrophiles (18). The drug-resistance spectrum of GST-π is cisplatin. However, the drug-resistance spectrum of P-gp is vincristine and doxorubicin (19,20). Therefore, the expression of P-gp and GST-π should be taken into consideration when designing clinical trials.

It has been previously suggested that aromatic compounds can induce GST-π expression (21,22). In addition, it is well known that GBC is closely associated with gallstone and chronic cholecystitis, which may generate aromatic compounds due to the long-term stasis of bile and bacterial infection (23). Thus, we hypothesized that aromatic compounds are not only comprised of chemical factors for the carcinogenesis of GBC, but are also important for GST-π expression.

The main prognostic factors of GBC are the clinical or pathological stages (24) and the Nevin staging system for GBC is widely used (25). The Nevin stage of GBC is mainly defined according to metastasis and invasion. In the present study, P-gp and GST-π showed lower levels in non-metastatic tumors (Nevin stage, I, II and III) than in metastatic tumors (Nevin stage, IV and V), suggesting that P-gp and GST-π may be used as indicators for invasion and metastasis. In the present study, patients with positive P-gp or GST-π expression showed a shorter 2-year survival rate compared with patients with a negative expression. Furthermore, patients with coexpression of P-gp and GST-π were associated with the worst prognosis. These results demonstrate that P-gp and GST-π may be used as prognostic markers for GBC and were consistent with previous studies on liver, colon, breast and ovarian cancer (26–28).

A close correlation between GST-π and P-gp expression was also identified in the current study, with coexpression observed in 59.5% of patients with GBC. Similar studies have shown that the rate of coexpression was 93% in patients with leukemia and 80% in patients with lung cancer (29,30). It has been demonstrated there are abnormal expression of genes, such as c-erbB-2, neu, P53, ras, INT2, HSTF1, bcl-2, c-fos and c-jun in various types of cancer, including GBC, and these genes not only correlate with drug resistance but also frequently regulate and co-amplify P-gp and GST-π genes (28–34). Therefore, the higher expression levels of P-gp and GST-π in patients with GBC may be a reflection of the abnormal expression of oncogenes and cancer suppressor genes.

In conclusion, results of the present study suggest that P-gp is a prognostic marker for GBC. In the future, the detection of P-gp and GST-π in patients with GBC may contribute to chemotherapeutic and surgical decisions.

## Figures and Tables

**Table I:** Demographic and clinical data and expression of P-gp and GST-π of gallbladder carcinoma patients.

Parameter	Cases, n	P-gp staining			Positive rate, %	P-value	GST-π staining			Positive rate, %	P-value
	
		−	+	++			−	+	++		
Gender											
Male	16	3	11	2	81	-	6	8	2	63	-
Female	26	7	11	8	73	0.22	9	12	5	65	0.86
Age, years											
≥60	26	5	12	9	81	-	7	16	3	73	-
<60	16	5	10	1	69	0.11	8	4	4	50	0.10
Presence of gallstones											
Yes	20	5	8	7	75	-	7	9	4	65	-
No	22	5	14	3	78	0.21	8	11	3	64	0.85
Pathology											
Adenocarcinoma (NOS)	28	5	17	6	82	-	9	14	5	68	-
Papillary adenocarcinoma	5	2	2	1	60	-	3	2	-	40	-
Adenosquamous	3	1	2	-	67	-	2	1	-	33	-
Mucinous adenocarcinoma	2	-	1	1	100	-	-	1	1	100	-
Undifferentiated carcinoma	4	2	-	2	50	0.48	1	2	1	75	0.41
Histological grade											
I	9	4	4	1	56	-	4	5	-	56	-
II	19	3	12	4	84	-	6	10	3	68	-
III	14	3	6	5	79	0.35	5	5	4	64	0.46

P-gp, P-glycoprotein; GST-π, glutathione *S*-transferase π; NOS, not otherwise specified.

**Table II:** Logistic regression analysis of metastasis of gallbladder carcinoma.

Variable	Regression coefficent	Standard error	P-value
P-gp	3.09	1.53	0.044
Tumor grade	0.48	1.00	0.631
Age	0.03	0.08	0.705
Gender	2.61	1.69	0.123
Pathological type	0.55	1.59	0.727
Gallstones	2.41	1.71	0.158

P-gp expression was coded as: 1, postive; 2, negative. Tumor grade was coded 1–3, with increasing grade. Gender was coded as: 1, male; 2, female. Pathological type was coded as: 1, adnocarcinoma; 2, non-adnocarcinoma. Presence of gallstones was coded as: 0, no; 1, yes. P-gp, P-glycoprotein.

**Table III:** Logistic regression analysis of 2-year survival of gallbladder carcinoma patients.

Variable	Regression coefficent	Standard error	P value
P-gp	−2.76	1.47	0.061
Tumor stage	0.84	2.13	0.693
Tumor grade	−1.28	0.90	0.153
Age	0.01	0.06	0.959
Gender	−0.46	1.15	0.687
Pathological type	0.16	1.19	0.896
Gallstones	0.05	1.18	0.969

P-gp expression was coded as: 1, postive; 2, negative. Tumor stage was coded as: 1, I+II+III; 2, IV+V; according to Nevin staging. Tumor grade was coded, 1–3 with increasing grade. Gender was coded as: 1, male; 2, female. Pathological type was coded as: 1, adnocarcinoma; 2, non-adnocarcinoma. Presence of gallstones was coded as: 0, no; 1, yes. P-gp, P-glycoprotein.
